# Abnormal T-Cell Development in the Thymus of Non-obese Diabetic Mice: Possible Relationship With the Pathogenesis of Type 1 Autoimmune Diabetes

**DOI:** 10.3389/fendo.2018.00381

**Published:** 2018-07-12

**Authors:** Daniella A. Mendes-da-Cruz, Julia P. Lemos, Geraldo A. Passos, Wilson Savino

**Affiliations:** ^1^Laboratory on Thymus Research, Oswaldo Cruz Institute, Oswaldo Cruz Foundation, Rio de Janeiro, Brazil; ^2^National Institute of Science and Technology on Neuroimmunomodulation, Oswaldo Cruz Institute, Oswaldo Cruz Foundation, Rio de Janeiro, Brazil; ^3^Department of Morphology, Physiology and Basic Pathology, Ribeirão Preto Medical School, School of Dentistry of Ribeirão Preto, University of São Paulo, Ribeirão Preto, Brazil; ^4^Department of Genetics, Ribeirão Preto Medical School, University of São Paulo, Ribeirão Preto, Brazil

**Keywords:** non-obese diabetic mouse, type 1 diabetes, thymus, autoimmune diabetes, insulin, insulin-like growth factor, miRNA

## Abstract

Type 1 diabetes (T1D) is an autoimmune disease caused by the destruction of insulin-producing cells in the pancreas, by direct interactions with autoreactive pancreas infiltrating T lymphocytes (PILs). One of the most important animal models for this disease is the non-obese diabetic (NOD) mouse. Alterations in the NOD mouse thymus during the pathogenesis of the disease have been reported. From the initial migratory disturbances to the accumulation of mature thymocytes, including regulatory Foxp3^+^ T cells, important mechanisms seem to regulate the repertoire of T cells that leave the thymus to settle in peripheral lymphoid organs. A significant modulation of the expression of extracellular matrix and soluble chemoattractant molecules, in addition to integrins and chemokine receptors, may contribute to the progressive accumulation of mature thymocytes and consequent formation of giant perivascular spaces (PVS) that are observed in the NOD mouse thymus. Comparative large-scale transcriptional expression and network analyses involving mRNAs and miRNAs of thymocytes, peripheral T CD3^+^ cells and PILs provided evidence that in PILs chemokine receptors and mRNAs are post-transcriptionally regulated by miR-202-3p resulting in decreased activity of these molecules during the onset of T1D in NOD mice. In this review, we discuss the abnormal T-cell development in NOD mice in the context of intrathymic expression of different migration-related molecules, peptides belonging to the family of insulin and insulin-like growth factors as well as the participation of miRNAs as post-transcriptional regulators and their possible influence on the onset of aggressive autoimmunity during the pathogenesis of T1D.

## Introduction

Type 1 diabetes (T1D) is a multifactorial disease caused by autoimmune destruction of pancreatic beta cells, which results in a breakdown of insulin production and glucose metabolism (1). The mechanisms involved in autoimmunity during the pathogenesis of T1D include factors of humoral immunity, such as the presence of circulating autoantibodies (anti-insulin, among other islet autoantibodies), that can be used as biomarkers of the disease (2–4). Mechanisms involving cellular immunity are evidenced by the presence of mononuclear cell infiltrates in the islets of Langerhans. CD8 T cells are the most predominant infiltrating-cells, followed by macrophages, CD4 T cells, B lymphocytes and plasma cells (5). In addition to the cellular infiltrate, the upregulation of MHC class I on β-cells may increase their susceptibility to T-cell–mediated killing (6). Most of the studies in humans were performed in pancreas samples removed post-mortem. Due to the limited availability concerning the samples and difficulties in studying the mechanisms of autoimmunity in humans, the use of experimental models are essential for studies on the pathogenesis of T1D. Among the available experimental models, the non-obese diabetic (NOD) mouse is particularly well characterized. They spontaneously develop the disease and present several characteristics that are similar to the pathogenesis of the human T1D (7, 8). Briefly, insulitis starts in general at 3 weeks of age in female mice, concomitantly with the appearance of initial thymic alterations, and the disease onset occurs at 10–12 weeks, depending on the colony. At this point, different alterations in the thymus have been described, as we discuss below. Nevertheless, before entering this discussion, it seems worthwhile to provide a basic background on some physiological aspects of the thymus, including the intrathymic T-cell differentiation, as well as production of hormones by thymic cells.

## The thymus and thymocyte development

The thymus is a primary lymphoid organ where T cells are generated. Inside the thymic tissue, precursor cells pass through distinct differentiation stages until becoming mature CD4 or CD8 single-positive (SP) thymocytes expressing the T-cell receptor (TCR), which are ready to emigrate to peripheral lymphoid organs and properly finish their maturation (9). Cell differentiation occurs in parallel with cell migration, so that the immature double-negative (DN) for the CD4^−^CD8^−^ co-receptors and double-positive (DP) CD4^+^CD8^+^ thymocytes are localized in the cortical region of the thymic lobules, while more mature CD4SP or CD8SP thymocytes are localized in the medulla (10). DP thymocytes express low amounts of TCR after gene rearrangement. This expression is increased during differentiation to TCR^high^ CD4SP or CD8SP cells. Differentiating cells undergo apoptosis if their TCR interact with high avidity with self-antigens coupled to major histocompatibility complex (MHC) class I or class II molecules expressed by microenvironmental cells in the thymus, in a process called negative selection. Alternatively, some clones that recognize self-antigens with high avidity become regulatory CD4^+^CD25^+^Foxp3^+^ T cells (Treg), a mechanism that seems to depend on TCR signaling avidity and duration, TGF-β-mediated survival and cytokines, such as IL-2, IL-7, and IL-15 (11, 12). These thymus-derived Treg cells account for the majority of Tregs in the periphery, compared with Tregs differentiated from conventional naïve T cells (13). Together, these processes avoid the development of self-antigen reactive cells and therefore prevents autoimmunity (14).

In the thymus, the expression of many peripheral tissue antigens (PTAs) in medullary thymic epithelial cells (mTEC) is regulated by the autoimmune regulator (AIRE) transcription factor (15). The PTAs are presented by MHC molecules and can induce negative selection of autoreactive thymocytes (16). The homozygous loss or mutations in the Aire gene cause the autoimune polyendocrinopathy–candidosis–ectodermal dystrophy (APECED) syndrome in humans, characterized by the development of autoimmune diseases including T1D in 10–20% of the cases (17–19). In mice, Aire disruption leads to immune cell infiltration in several organs and APECED-autoimmune like manifestations (15, 20).

While migrating through the thymic lobules, developing thymocytes also interact with other microenvironmental cells such as dendritic cells (DCs) and macrophages, as well as with extracellular matrix (ECM) molecules and soluble proteins such as cytokines, chemokines, growth factors and thymic hormones (thymulin and thymopoietin, for example). Other hormones produced by endocrine glands (growth hormone, glucocorticoids, prolactin, oxytocin and insulin) can also be produced locally, and play a role in the physiology of the thymus and the generation of the T-cell repertoire (10).

## Intrathymic expression of peptides and receptors from the insulin/IGF family

Insulin is a polypeptidic hormone produced as a pre-prohormone, the pre-proinsulin, which is processed to proinsulin that is cleaved, in turn, to mature insulin. Only pancreatic beta cells are capable to secrete mature insulin in response to glucose (21). Despite that, proinsulin gene is naturally expressed at low levels in fetal and postnatal thymi in humans, rats and mice (22). Although the expression of proinsulin in the thymus is not necessary for T cell differentiation and growth (23, 24), variations in the expression of the insulin gene in the thymus, but not in the pancreas, in both humans and mice, can modulate self-tolerance to insulin, with the expression levels being inversely correlated with T1D susceptibility (21, 25).

In humans, the insulin gene is under the control of a variable number of tandem repeats (VNTR) minisatellites, mapping 5′ to the insulin gene promoter. VNTR, commonly known as IDDM2 susceptibility locus, are extremely polymorphic regions both in size and sequence (25, 26), and three allele classes have been characterized: class I, composed by 20–63 repeats of the consensus unit ACAGGGGTCTGGGG, class II alleles containing 64–139 repeats and class III alleles containing 140 to >200 repeats. Although VNTR have little effect in pancreatic insulin transcription, class I alleles in the thymus correlate with low and class III with high levels of insulin mRNA (25, 27).

There is no VNTR regions in mice, but two nonallelic insulin genes Ins1 and Ins2 encoding proinsulin 1 and 2 respectively (28, 29). Both insulin genes are expressed by 1–3% of mTECs in the thymus under control of AIRE (15, 30, 31), but Ins2 expression is predominant in the thymus. Although suggested that this predominant expression leads to a higher tolerance to proinsulin 2 (32), it was demonstrated that proinsulin 2 expression leads to T cell tolerance to an epitope shared by both proinsulin 1 and 2 (33). The copy numbers of insulin gene in the mouse thymus inversely correlates to the numbers of insulin-specific autoreactive T cells in the periphery, so that mice expressing low levels of thymic insulin, (even though pancreatic insulin remains unaltered), present peripheral reactivity to insulin, whereas mice with normal thymic insulin expression have no significant response (34). This effect is transferable by thymic transplantation (35), showing that thymic insulin expression plays a critical role in thymic selection and T1D susceptibility.

Insulin-like growth factors (IGF) 1 and 2 are polypeptidic growth factors, members of the family of insulin-related peptides, produced in many tissues where they can play endocrine and paracrine functions (36). Both IGF-1 and 2 can bind to type 1 and 2 IGF receptors (IGF-1R/IGF-2R) with high affinity and to insulin receptors (INS-R), with low affinity (37). All the genes of the insulin family are expressed in the thymus during the fetal life; IGF2 is predominantly expressed in the rat, mouse and human thymi by TEC and Thymic Nurse Complexes (TNC), followed by IGF1, expressed by TECs and macrophages. The proinsulin genes are expressed by mTECs and DCs (38–41). In general, protein levels are related with gene levels in the case of these molecules.

After birth, IGF-2 gene expression and protein levels decrease and reach the same levels of IGF-1 (32). IGF-2 participates both in T cell development and negative selection (42). Studies using fetal thymic organ cultures (FTOC) demonstrated that the blockage of IGF-mediated signaling between TEC and thymocytes inhibits early T cell proliferation and differentiation (23). Specific anti-IGF-1 antibodies treatment lead to a decreased DN relative cell numbers while the inhibition of IGF-2, IGF-1R or IGF-2R impaired differentiation from the DN to the DP stage. The same study showed a decrease in total T cell numbers under treatment with anti-IGF-1R and anti-IGF-2R antibodies. Moreover, transgenic IGF-2 expression resulted in abnormalities in the terminal differentiation and increased proliferation of TECs. The deposition of fibronectin and laminin is enhanced in human TEC cultures and in the thymus of IGF-2 transgenic mice, in parallel with the enhancement of thymocyte adhesion to TEC monolayers and thymocyte migration (43).

IGF-2 expression by the thymic epithelium is under control of AIRE, and the IGF-2 gene is located adjacent to the Ins gene (44, 45). Its predominant expression among insulin family members could be explained by IGF-2 close homology to the other members with high conserved peptides sequences of the family. This could lead to the development of tolerance to IGF-2 and related molecules, including insulin (45). Igf-2^−/−^ mice present weaker tolerance to insulin when compared with wild type animals and the production of specific antibodies to IGF-2 is more difficult than to IGF-1 or insulin (46–48).

IGF-1 and its receptor are implicated in several growth hormone (GH) effects in the thymus, as TEC proliferation and thymocyte/TEC adhesion (49), as we further discuss below.

## GH/IGF-1 axis in the thymus

Growth hormone is a member of a family of growth factors that includes prolactin and other hormones. It is produced and stored mainly in the anterior pituitary under control of hypothalamic hormones, as the GH-releasing hormone, hypothalamic GH release-inhibiting factor and somatostatin (50), although the production by other cell types was observed, including leukocytes and TECs (51). The early experiments showing that GH is thymotropic revealed that GH-deficient mice present thymus atrophy and this effect is also observed after GH anti-serum treatment of mice with intact pituitary (52).

The GH receptor (GHR) is expressed in cortical and medullary TECs (53, 54) as well as in thymocytes (51, 55), and plays a role in thymic function and T-cell differentiation. The decline of GH production is related with thymic involution (56). Moreover, transgenic mice overexpressing GH have an enlarged thymus, as well as mice and humans treated with recombinant forms of the hormone (57). GH can also modulate the thymic microenvironment by increasing the secretion of cytokines, chemokines and thymulin, consequently modulating thymocyte adhesion and migration (57–60).

As mentioned above, some of the GH effects in the thymus are mediated by IGF-1. Murine TEC lines treated with GH or IGF-1, present an enhancement in ECM molecules production as type IV collagen, fibronectin and laminin, besides the expression of the integrins VLA-5 (alpha 5 beta 1 integrin, a fibronectin receptor) and VLA-6 (alpha 6 beta 1 integrin, a laminin receptor). Treatment with GH also augmented the thymocyte/TEC adhesion, a phenomenon that was blocked by anti-IGF-1 and anti-IGF-1R antibodies (61).

Since the interactions of thymocyte and TECs are crucial for thymocyte development and thymus physiology, one can argue that together, the GH/IGF-1 axis, besides IGF-2 and insulin can shape the T-cell repertoire. Moreover, it is conceivable that defects in the negative selection against PTAs related to this family might cause autoimmunity, as for example T1D, in the case of insulin-related peptides expressed intrathymically.

## Thymic alterations in NOD mice

Several morphological and phenotypic alterations are observed in the NOD mouse thymus. The most evident is the formation of giant perivascular spaces (PVSs), which are filled with mature CD4SP and CD8SP cells, B cells and regulatory Foxp3^+^ cells (62–64). We have described that cells inside giant PVSs present a defect in the membrane expression of the integrin-type fibronectin receptor VLA-5 (CD49e/CD29) that may lead to their accumulation and retention in the thymus (65). The formation of giant PVSs also changes the TEC network and ECM contents, both inside PVS and in the thymic parenchyma. Particularly, there is an important deposition of fibronectin inside these spaces (Table 1).

**Table 1:** Alterations observed in the NOD mouse thymus.

	Alteration^*^	**Affected compartments**	**References**
**THYMIC PARENCHYMA STRUCTURES**			
PVS	↑↑↑	Medullary region	(62)
TECs	↓	Medullary region	(62)
**CELL MIGRATION-RELATED MOLECULES**			
VLA-5	↓↓↓	Mainly CD4SP CD8SP and Foxp3^+^ regulatory T cells	(64, 65)
VLA-4	↑	DP, CD4SP and CD8SP thymocytes	(64, 65)
VLA-6	↑↑↑	All thymocyte subpopulations	(64)
CXCR4	↓	CD8SP thymocytes	(64)
CXCL12	↑	Mainly inside giant PVSs	(64)
Fibronectin	↑	Mainly inside giant PVSs	(64, 65)
Laminin	↑	Mainly inside giant PVSs	(64, 65)
Type I and IV collagens	↑	Mainly inside giant PVSs	(62)
**INSULIN FAMILY-RELATED PEPTIDES AND RECEPTORS**			
Insulin	↓↓	mTECs	(66)
IGF-1, IGF-2, INS-R, IGF-1R, IGF-2R	ND	–	–
GH, GHR	ND	–	–
**miRNAs**			
miR-19a	↓/–	Thymocytes TCR^+^/NKT17	(67)
miR-19b	↓/–	Thymocytes TCR^+^/NKT17	(67)
miR-133b	–/↑↑	Thymocytes TCR^+^/NKT17	(67)
miR-124a	↑/–	Thymocytes TCR^+^/NKT17	(67)
miR-326	↓/–	Thymocytes TCR^+^/NKT17	(67)

* *Alteration comparing NOD with other inbred mouse strains; PVS, perivascular space; TEC, thymic epithelial cell; IGF, insulin-like growth factor; INS-R, insulin receptor; IGF-R, insulin-like growth factor receptor; GH, growth hormone; GHR, growth hormone receptor; NKT17, IL-17-producing natural killer T cells; ND, not described*.

The accumulation of thymocytes and enlargement of PVS starts to be observed at 4 weeks of age in female mice, which are more susceptible for T1D. Clear-cut giant PVS are observed in pre-diabetic mice (9–12 weeks of age), that already present insulitis (62).

*Ex vivo* functional assays revealed that NOD thymocytes have a defect in the migratory capacity toward fibronectin, but not laminin. Interestingly, migration toward the chemokine CXCL12 is enhanced, and a synergic effect is observed when CXCL12 is combined with ECM molecules. In the case of fibronectin combined with CXCL12, despite the synergic effect, migration of NOD thymocytes is reduced compared with controls (65, 64).

**Figure 1:**
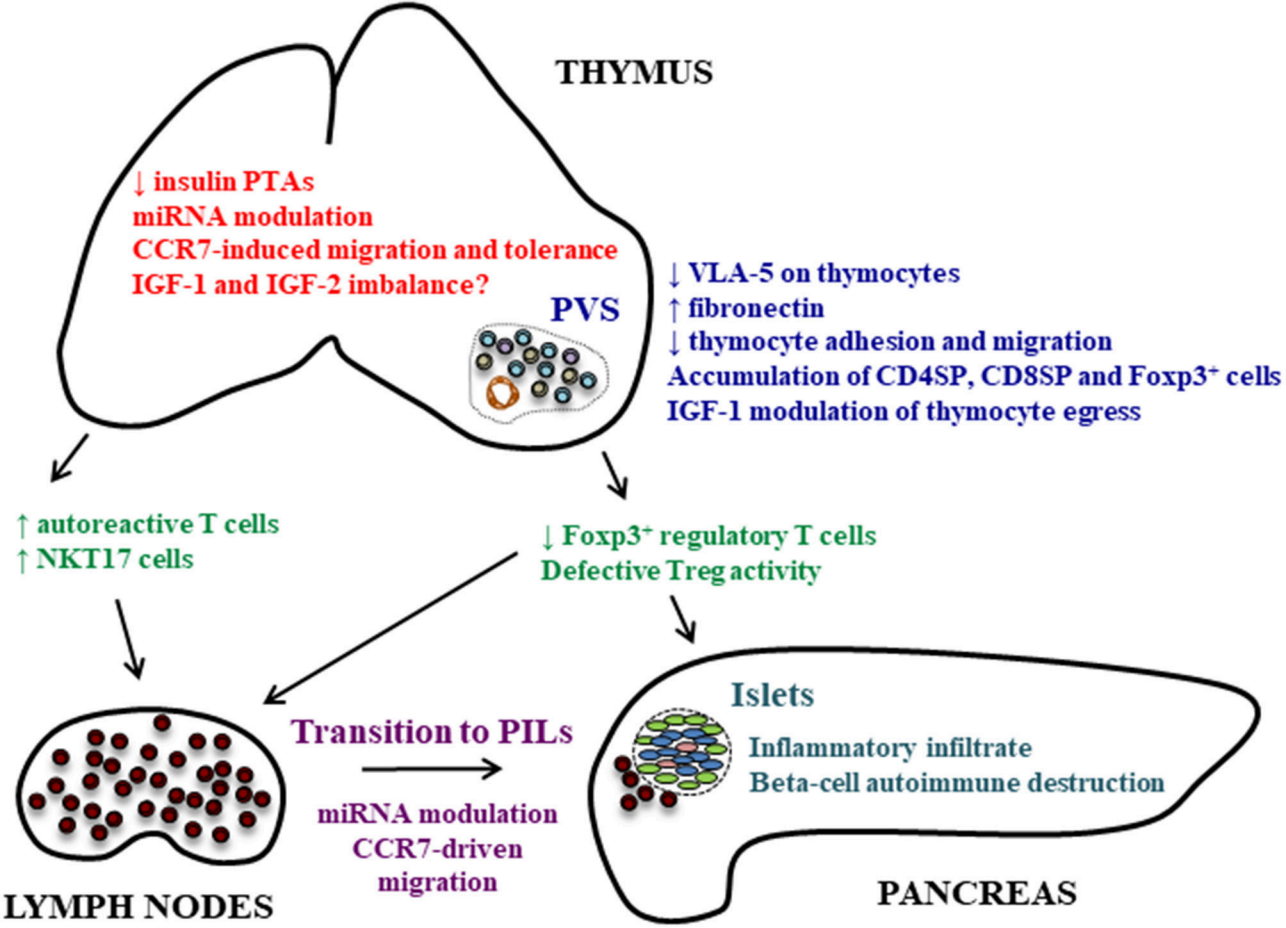
Thymic alterations that can play a role in autoimmune pathogenesis of T1D. Different thymic alterations are observed in the NOD mouse thymus concerning the expression and role of molecules involved in cell adhesion and migration, peptidic hormones under control of the AIRE gene and miRNAs. The diminished insulin expression in the thymus and miRNA modulation of PTAs can lead to the generation of autoreactive cells. The defect in VLA-5 membrane expression on thymocytes and modulation of chemokine receptors are related with the accumulation of thymocytes, including Foxp3^+^ regulatory cells, and the formation of fibronectin-enriched giant PVSs. This accumulation of thymocytes may also be modulated by IGF-1, and can explain the reduced Treg numbers in peripheral lymphoid organs and pancreas affecting the balance between Tregs and effector T cells, although this is still controversial. Tregs can also present defective activation. In peripheral lymphoid organs, the transition of T cells to PILs is under control of miRNAs that can modulate the expression of chemokine receptors and consequent migration of these cells to the pancreas. Together, these changes may possibly be due to intrathymic hormonal imbalance, comprising the expression of insulin, IGF-1 and IGF-2.

Another experimental strategy trying to understand the role of VLA-5 in thymocyte accumulation in giant PVS in NOD mice comprised ECM-transmigration assays, which mimic the migration of thymocytes through fibronectin-enriched PVSs and then the transmigration through endothelium. These experiments revealed that NOD thymocytes that first encounter fibronectin molecules transmigrate less then controls (64). Conversely, differences in transmigration assays were not observed when laminin was applied, reinforcing the concept that VLA-5/fibronectin interactions can play a role in the accumulation of thymocytes in PVS during the pathogenesis of T1D (Figure 1).

As mentioned above, thymic insulin expression plays a role in thymic selection processes and T1D development. The expression levels of insulin genes are also altered in the NOD mouse thymus. The Ins2 gene expression is normal at 2 weeks of age but become lower at 3 weeks, which may favor loss of tolerance to insulin in NOD mice (68, 66). Moreover, Ins2^−/−^ NOD mice have accelerated insulitis and autoimmune diabetes onset in females, increased disease in males, with enhanced prevalence of insulin autoantibodies and stronger insulin response (33). Conversely, insulitis and diabetes onset were delayed in NOD Isn1^−/−^ mice, which can be explained by the dominance of the Ins2 gene in the thymus, whereas Ins1 is more prominent in pancreatic beta cells (69). Prevention of both insulitis and diabetes can be seen in transgenic NOD mice expressing increased levels of Ins2 under the MHC class II promoter (70), and also after intrathymic administration of insulin (71).

The expansion of autoreactive T cell clones in NOD mice can also be affected by proinsulin gene expression. Although the numbers of CD4SP and CD8SP thymocytes do not change, proinsulin-1 or−2 deficiency in NOD mice causes changes in the T-cell repertoire generated in the thymus and peripheral lymphoid organs, and is associated with a significant expansion of insulin–reactive CD8SP T cell clones in the pancreatic draining lymph node (72).

The effects of IGF-1 on cell trafficking were also analyzed in the adoptive T cell transfer model in NOD mice (73). T cells from diabetic NOD Thy-1.2 mice were injected into congenic NOD Thy-1.1 mice. In this model, reconstitution of the thymus of irradiated recipients with donor cells was not influenced by IGF-1 treatment, but the percentage of donor T cells was significantly reduced in the spleen of IGF-1 treated mice in contrast to the thymus, suggesting that IGF-1 could influence T cell trafficking from the thymus to peripheral lymphoid organs. This might be due to effects of IGF-1 upon the Sphingosine kinase/sphingosine-1-phosphate axis, as demonstrated for myoblast differentiation (74).

The role of IGF-2 specifically in the thymus of NOD mice is not yet defined. However, transcriptome studies revealed that IGF-2 mRNA is downregulated when comparing mTECs from newborn and 5 week old NOD mice (75). A downmodulation was also observed for Ins1 and Ins2 mRNA expression. Interestingly, the same was observed in BALB/c mice for both IGF-2 and Ins-2, but not for Ins-1 mRNA. Moreover, the reconstruction of post-transcriptional miRNA-mRNA interaction networks revealed that some miRNAs, including the miR-647 that targets IGF-2 mRNA, were included in the network of BALB/c, but not in the NOD mice (75). In this context, since the expression of PTA mRNAs (and the respective proteins) in mTECs is important for the negative selection process, the mechanisms that inhibit the regulatory action of miRNAs may be acting in these cells.

The expression of other miRNAs is altered in the thymus of NOD mice when comparing with C57BL/6 mice (Table 1). The miR-19a, miR-19b and miR-326 are downregulated whereas miR-124a is upregulated on TCR^+^ thymocytes. MiR-133b is upregulated only in natural killer T (NKT) cells in the thymus (67). This miRNA targets and regulates the transcription factor Th-POK, which negatively regulates the differentiation of IL-17 producing NKT cells (NKT17). Thus, the diminished expression of Th-POK can induce the differentiation of NKT17 cells and explain the enhanced numbers of these cells in the thymus and peripheral lymphoid organs of NOD mice (67), which can be related with exacerbation of diabetes (76).

## The role of miRNAs throughout transition of thymocytes into pancreas infiltrating lymphocytes

During the period of evolution of autoimmune reactivity in NOD mice, even before the appearance of clinical signs of T1D, thymocytes that differentiate into peripheral CD3^+^ T lymphocytes sequentially modulate (up- or down-regulate), a significant set of mRNAs that encode proteins involved in the intrathymic negative selection, T cell maturation, differentiation and autoreactivity (77). Among the peripheral T lymphocytes residing in the spleen and/or in the lymph nodes some autoreactive clones will evolve into PILs in mice and humans (1, 78, 79). Insulin-specific CD4 and CD8 T cells targeting multiple epitopes are predominant in the islet infiltrating T cells in pre-diabetic NOD mice pancreas (80), with proinsulin 2 being proposed as the major isoform recognized by those cells (33).

During the transition of peripheral T lymphocytes into PILs, a large set of mRNAs is transcriptionally modulated causing changes in the transcriptome profile of these cells with parallel modulation of miRNAs. These transcriptional changes are robust enough to hierarchize the different cell types (thymocytes, CD3^+^ peripheral T lymphocytes and PILs) and the different stages of NOD mice regarding the onset of T1D (pre- or diabetic animals) according to their respective mRNA or miRNA expression signatures (81). The miRNA modulation strongly suggests that transition into PILs would be under posttranscriptional control, i.e., the effect of specific miRNAs upon target mRNAs that encode proteins involved in this process.

The reconstruction of miRNA-mRNA interaction networks, based on differential expression profiling of peripheral T lymphocytes during their transition into PILs in NOD mice predicted mRNA targets in an unbiased way. As these cells develop into CD3^+^ peripheral T cells and then into PILs, thymocytes exhibited miRNA interactions with mRNA targets that encode proteins related to apoptosis, cell adhesion, positive and negative selection in the thymus. The interactions involving miR-202-3p with CCR7 mRNA were highlighted in the work of Fornari et al. (81), showing that CCR7 is involved with the control of central tolerance and mice lacking this chemokine receptor generated autoreactive T cells (82). Moreover, CCR7 directs T-cells toward the pancreas of NOD mice, since desensitization of CCR7 blocked T-cell migration from the bloodstream into pancreatic islets (83).

A second interaction emphasized was miR-202-3p-CD247 mRNA in NOD mice (81). Under disturbance during TCR signaling, the CD3 zeta chain enhanced autoimmune diabetes in mice (84).

The evidence at this moment suggests that the transition into PILs is under post-transcriptional control exerted by miRNAs (Figure 1). Interestingly, some miRNAs such as miR-375, miR-30d and miR-9, can control insulin synthesis and secretion by pancreatic beta-cells (85) in NOD mice. Whether miRNAs also regulate the intrathymic production of proinsulin/insulin and IGF remains unknown.

## Future developments and concluding remarks

Although the precise biological mechanism(s) underlying how differentiating thymocytes evolve to autoreactive T-cells infiltrating and destroying pancreatic beta cells are not elucidated, it is likely that disturbances of gene and miRNA signatures may be part of this process, as well as changes in the profiles of cell migration of both thymocytes and peripheral T lymphocytes. In this context, besides the questions raised throughout the text, other important questions remain unanswered, such as the possible direct role of the thymic alterations in the pathogenesis of T1D and the presence of similar alterations in humans.

In humans, serum GH levels are enhanced in T1D patients (86), and IGF-1 and IGF-1R mRNA levels are reduced in peripheral blood mononuclear cells (87). The circulation levels of GH are enhanced whereas IGF-1 levels are diminished in NOD diabetic mice 4 weeks after the appearance of glycosuria (88), suggesting similarities in hormone imbalance between T1D patients and NOD mice at least after disease diagnosis.

Hormonal imbalance in the thymus can be involved in the control of the physiology of the organ in NOD mice and humans, as the properly maturation of the cells, cell adhesion, migration, accumulation and egress, by the modulation of ECM molecules and integrins, chemokines and chemokine receptors, sphingosine-1-phosphate and sphingosine-1-phosphate receptor 1 (10, 64, 65, 89). As an example, GH/IGF-1 axis can modulate the expression of cytokines, chemokines and ECM molecules and receptors in the thymus (61). GH modulates thymocyte adhesion and migration properties, and promotes thymocyte egress (59). The effects of GH can be regulated by IGF-1, which can in turn bind IGF-R and insulin receptor (90). Lower insulin levels in the thymus are related with reactivity to insulin in the periphery, including in NOD mice (33). Together, these mechanisms can shape the T cell repertoire and change the frequency of Tregs and the ratio of Treg and effector T cells (34, 45). The diminished frequency of Tregs in NOD mice is controversial, and most studies in T1D patients have reported no differences in the frequency of Tregs in peripheral blood. Likewise, phenotype and diminished suppressive capacity have been reported in both NOD and T1D patients (64, 91–93). Whether these specific issues are related with hormonal imbalance during the pathogenesis of T1D, comprising the expression of insulin, GH/IGF-1 and IGF-2, need further investigation.

## Author's note

This manuscript is dedicated to Prof. Mireille Dardenne, for her significant contribution in the field of Immunoendocrinology along the last 50 years. In 2017 we celebrated her 80th birthday.

## Author contributions

All authors listed conceived and wrote the manuscript, and approved it for publication.

### Conflict of interest statement

The authors declare that the research was conducted in the absence of any commercial or financial relationships that could be construed as a potential conflict of interest. The handling Editor is currently co-organizing a Research Topic with the authors DMC, WS and confirms the absence of any other collaboration.
